# Freshwater macroinvertebrate samples from a water quality monitoring network in the Iberian Peninsula

**DOI:** 10.1038/sdata.2018.108

**Published:** 2018-06-05

**Authors:** Nora Escribano, Javier Oscoz, David Galicia, Tommaso Cancellario, Concha Durán, Patricia Navarro, Arturo H. Ariño

**Affiliations:** 1 Universidad de Navarra, Facultad de Ciencias, Departamento de Biología Ambiental, Pamplona 31008, España; 2 Servicio de Calidad de Aguas, Confederación Hidrográfica del Júcar, Avda. Blasco Ibáñez 48, Valencia 46010, España; 3 Área de Calidad de Aguas, Confederación Hidrográfica del Ebro, Paseo de Sagasta 24-28, Zaragoza 50071, España

**Keywords:** Biodiversity, Freshwater ecology, Hydrology, Limnology

## Abstract

This dataset gathers information about the macroinvertatebrate samples and environmental variables collected on rivers of the Ebro River Basin (NE Iberian Peninsula), the second largest catchment in the Iberian Peninsula. The collection is composed of 1,776 sampling events carried out between 2005 and 2015 at more than 400 sampling sites. This dataset is part of a monitoring network set up by the Ebro Hydrographic Confederation, the official body entrusted with the care of the basin, to fulfill the requirements of the European Water Framework Directive. Biological indices based on the freshwater macroinvertebrate communities were used to evaluate the ecological status of the water bodies within the basin. Samples were qualitatively screened for all occurring taxa. Then, all individuals from all taxa in a quantitative subsample of each sample were counted. Biological indices were calculated to estimate water quality at each sampling site. All samples are kept at the Museum of Zoology of the University of Navarra.

## Background & Summary

Freshwater ecosystems, a mere ten-thousandth of the world’s aquatic ecosystems by surface, harbour at least 100,000 species. Freshwater bodies are also an indispensable resource for human communities^1^. Yet, it is argued that these ecosystems are the most endangered ones in the world, threatened by overexploitation, water pollution, habitat degradation, invasion by exotic species, and flow modification^1^. Managing and conserving these valuable ecosystems has been central to environmental policy over the last decades. Within Europe, concern about the status of European waters was finally addressed on October 23rd, 2000 when the Directive 2000/60/EC of the European Parliament and the Council was passed, establishing the Water Framework Directive (hereinafter WFD)^2^. WFD established the river basins as the units for managing water systems because they constitute geographical units with clear natural limits. The assessment of the ecological status of waters was entrusted to a broad range of approaches including traditional chemical analysis and much more integrative analyses based on biological indices^3^.

Nearly a century ago, the Spanish Government had already encouraged the constitution of organisms that would manage the water resources in the country. As in the WFD, the river basin unit was chosen as the best model to manage waters. The Ebro Hydrographic Confederation (“*Confederación Hidrográfica del Ebro*”, hereinafter CHE), was the first organism established in 1926. Since its constitution, the CHE has been developing hydrological plans, economic analyses of the use of water and monitoring networks on water quality within the Ebro River Basin, the second largest of the Iberian Peninsula, occupying most of Northeast Spain.

As part of the monitoring activity of the quality of the superficial water bodies (rivers, streams, and lakes), CHE implemented several networks based on chemical and biological analyses. In 2005 all these networks began to be called Control of the ecological status of the surface water bodies in the Ebro Basin [*Control del Estado de las Masas de Agua Superficiales*, hereinafter CEMAS]^4^. This development, involving changing the previous monitoring network designs, addressed the necessity to fulfill requirements and new guidelines of the WFD^5,6^ so that the data obtained could be comparable to other networks in Europe.

One of these networks focuses on sampling freshwater macroinvertebrates. This taxonomic group has been used in biotic indexes (e.g., Saprobic System, Biological Monitoring Working Party) worldwide since the early 1900’s to assess the water quality of rivers^7^. Macroinvertebrates constitute heterogeneous and diverse communities spanning several phyla that respond swiftly to different types of pollutants in a rather specific manner and are easy to collect and identify at least to family level. In addition, they are abundant and widely distributed all over the world’s freshwater ecosystms^7^. These characteristics make them good integrators of environmental conditions, enabling their convenient use for ecological monitoring.

We introduce the dataset *Macroinvertebrate samples from the water quality monitoring network along the Ebro Basin* that contains the information of the samples collected on rivers and streams of the Ebro River Basin (NE Iberian Peninsula). The collection is composed of 1,776 sampling events carried out between 2005 and 2015 at more than 400 sampling sites. These samples are all deposited at the Museum of Zoology (hereinafter MZNA) of the University of Navarra (https://www.unav.edu/web/museodecienciasnaturales). This dataset is part of the Freshwater samples in the MZNA-INV-FRW collection. The MZNA, an Open Access facility that discloses its collection data to the public, committed to preserve these samples as a valuable scientific asset that can be revised, verified and re-used in coming years^8^.

## Methods

### Study Area

**Figure 1:**
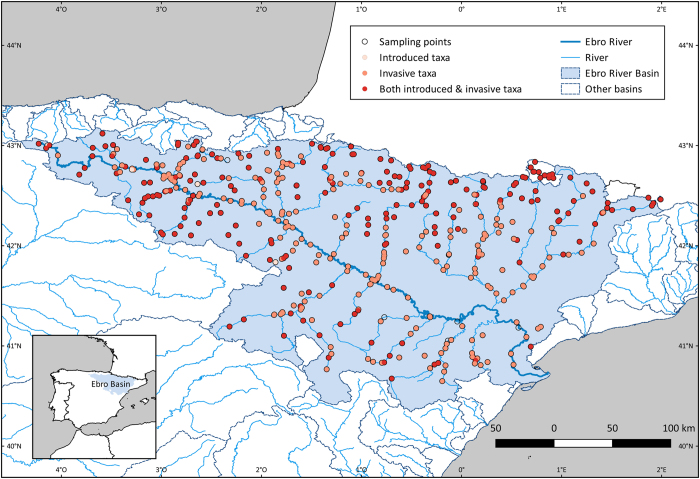
Map of the sampling points in the Ebro River Basin within the dataset *Macroinvertebrate samples from the water quality monitoring network along the Ebro Basin*.

The Ebro River Basin, located in the Northeast of Spain (Western Europe), drains an area of 85,362 km^2^ (Fig. 1). It extends from the western Pyrenees to the south of the Iberian Mountains, discharging into the Mediterranean Sea. Its main 702 tributaries run for an accumulated 12,000 km. The prevailing climate of the basin is Mediterranean with an average yearly precipitation of 620 mm corresponding to the Csb and Csa categories in the Köpen Climate Classification system^9^. However, the upper region of the basin is influenced by the Atlantic Ocean being a temperate climatic zone (Cfb and Cfa categories). Finally, the Pyrenean part of the basin presents a cold climate with fresh summers in the areas of higher altitude (Dfc and Dfb categories)^9^. The basin hosts a large number of ecosystems from the head of the Ebro River to its mouth. Eurosiberian communities (beech, grasslands) dominate the highest part of the river whereas Mediterranean ones take over in the middle region until the mouth^10^. The water uses of the basin are principally urban water supply, livestock, farming, and industry. However, the highest water consumption comes from agriculture (4,574 hm^3^/year) dedicated to 906,000 ha of irrigated crops^10^.

### Sampling design

The current monitoring network was designed in 2005 according to the criteria set up by the WFD, superseding the previous network where macroinvertebrate samplings to evaluate the water quality had been conducted by CHE. The new design enabled subsequent data to be compared to other monitoring networks.

Samplings were carried out annually from late spring to early autumn. Samplings could be postponed in the case of unfavorable environmental conditions such as heavy rainfall in elevated areas (e.g., the Pyrenees). Likewise, in the event of floods, samplings were conducted 15 days after the incident. Temporary streams were sampled when the conditions were optimal, that is, in the presence of running water^11^.

A total of 473 sites belonging to the CEMAS network were sampled from 2005 to 2015 (Fig. 1). Each sampling site covered a representative 100 m segment of the river having the essential habitats of that river’s stretch. The following features were taken into account for selecting a segment:

- The presence of rapid-slow running water.
- Fluvial morphology. For example, natural courses were chosen over channeled water.
- Vegetation coverage. Shady areas were avoided if they were not characteristic of the stretch.
- Areas near bridges and weirs were avoided unless they were representative of the stretch.
- Accessibility. Sampling sites were accessible and crossable.

At each sampling site, five types of habitats were taken into account: hard substrates, plant debris, bank bordered by vegetation, submerged macrophytes (if present), sand and other fine sediments. Once identified the microhabitats, the sampling effort (kicks) was distributed proportionally to the area of each of the microhabitats in the section. As a rule, the sampling effort consisted on twenty kicks. Macroinvertebrates were collected using a hand-net (25 cm×25 cm aperture, 500-μm-mesh size). In each sampling unit (kick), the substrate was removed 0.5 m in front of the mouth of the net (oriented against the flow). The final sampled area resulting from the twenty kicks was approximately 2.5 m^2^. Samples were fixed in 4% buffered formaldehyde, stored in plastic sealed jars, labeled and brought to the laboratory.

Simultaneously, electrical conductivity (precision 1 μS/cm), dissolved oxygen (precision 0.01 mg/L), temperature (precision 0.1 °C) and pH were measured using a multi-parameter water quality monitoring system (WTW Multi 340i) at each sampling site. Each instrument was calibrated daily during the sampling period.

### Laboratory work

Samples were taken to the laboratory for analysis. Each sample was sieved through 5 mm, 1 mm and 0.5 mm mesh sieves. The material was washed with abundant running water, separating the organisms from the remaining debris, gravel, and sand. Finally, the sample was divided into the respective fractions of the sieves and analyzed. In the 5 mm fraction, all organisms were counted and identified to the taxon level required by the *Iberian Biomonitoring Water Party* (IBMWP) index. Similarly, organisms from 1 mm and 0.5 mm fractions were identified to the taxon level required for the IBMWP index, but samples were divided into subsamples (e.g., one-quarter size) and only organisms from one of the subsamples were counted. This procedure allowed estimating the total abundance of each taxon from the subsample counts. The remaining sample (e.g., the uncounted three-quarters of the sample) was examined for families not caught in the subsample, but organisms were not counted. After processing the samples, the IBMWP and the *Iberian Average Score per Taxon* (ASPT) scores were calculated^12^. See the sampling protocol published by the Ministry of Agriculture, Food and Environment in 2013 (ref. 13) for further details.

### Data management and standardization

All the material from the samples was stored in plastic jars with 70% ethyl alcohol, labeled and stored at the MZNA facilities. Field data was digitized and incorporated into the MZNA database^14^. Unique accession numbers were assigned to each sample and each occurrence record.

The information about samples and occurrences was retrieved from the MZNA database and made fully compliant with the Darwin Core (DwC) standard (http://rs.tdwg.org/dwc/index.htm) by mapping all fields to 64 Darwin Core Terms (see Supplementary File 1 for further details). The resulting dataset was published through the Global Biodiversity Information Facility (GBIF: https://www.gbif.org/). Environmental data relevant to each sampling event were obtained from the CHE portal (http://www.chebro.es/).

## Data Records

The *Macroinvertebrate samples from the water quality monitoring network along the Ebro Basin* dataset is hosted by GBIF and can be downloaded as one single DwC-compliant.txt file (Data Citation 1). The data descriptor we present here corresponds to version 2.5 of this dataset.

The collection is a sample-based dataset, a recently launched extension of GBIF^15^, which contains 1,776 records of freshwater macroinvertebrates samples collected in the Ebro River Basin. These samples are stored in the MZNA facilities and are available to interested researchers on request.

The dataset also includes information about 266,400 presence/absence records of freshwater macroinvertebrates within the samples, and measurements of four environmental variables (electrical conductivity, dissolved oxygen, temperature, and pH) for 1,775 samples.

All samples are georeferenced as decimal degrees to 1/10000th of degree and dates are resolved to day.

**Figure 2:**
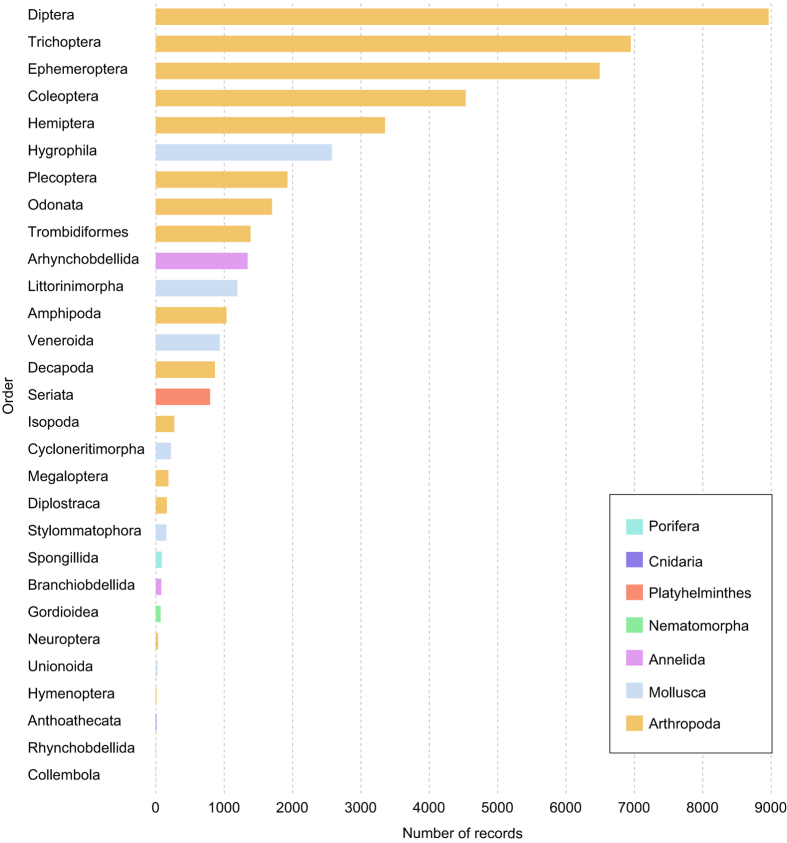
Number of records per order within the dataset. Only presence records are represented. Phyla Bryozoa and Nematoda are not represented as specimens were not classified to Order level or lower.

The collection comprises 150 taxa recorded in the Ebro River Basin belonging to 10 genera, 141 families, 31 orders, 15 classes and 9 phyla. Due to the identification protocol, not all the specimens were identified to the same taxon rank. Arthropoda is the most represented phylum in the dataset. It includes 218,448 records belonging to 18 orders, with Diptera, Coleoptera, and Trichoptera being the most abundant (Fig. 2). Two taxa are introduced, and four are invasive in the Iberian Peninsula. Within the phylum Mollusca, specimens belonging to the genus *Ferrisia* and family *Physidae* are introduced whereas genera *Corbicula* and *Dreissena* are invasive. Regarding arthropods, the genera *Pacifastacus* and *Procambarus*, both North American crayfish, are invasive in the Iberian Peninsula.

## Technical Validation

The main specimen identification was carried out by one of the authors (JO) using suitable literature^16,17^. Scientific names were validated according to the GBIF Backbone Taxonomy^18^. All samples were fully screened for the entire range of potential taxa required for the IBMWP index. Taxon absences recorded in the dataset are thus true absences as regards to the taxa listed in the IBMWP indices. Moreover, the dataset also includes information about several taxa not included in the IBMWP index taxon reference list: 1) phyla Bryozoa and Nematoda, 2) classes Maxillopoda (subclass Copepoda) and Arachnida, 3) order Diplostraca (suborder Anomopoda), 4) families Spongillidae, Gordiidae, Branchiobdellidae, Haemopidae, Succineidae, Chaoboridae, Hebridae, Ichneumonidae, Muscidae, Niphargidae, Osmylidae, Pediciidae, Sisyridae, 5) genera *Hydra*, *Podura*, *Corbicula*, *Dreissena*, *Pacifastacus*, and *Procambarus*.

The dataset was standardized to the Darwin Core standards. Guidelines by Chapman (2005)^19^ were followed to check for taxonomical, geographical and temporal errors in the dataset. Coordinates in UTM/MGRS were transformed to the geographic system. The consistency of all records was inspected by overlapping sampling site coordinates with a map of the Ebro Basin^10^. Collecting dates format was transformed to the ISO 8601 format (i.e., YYYY-MM-DD).

The entire process of debugging the dataset was done with R version 3.3.2 (R Core Team, 2016). Packages used were rgdal^20^, sp^21^ for geographic data and reshape2 (ref. 22) for handling with the dataset.

## Additional information

**How to cite this article**: Escribano, N. *et al*. Freshwater macroinvertebrate samples from a water quality monitoring network in the Iberian Peninsula. *Sci. Data* 5:180108 doi: 10.1038/sdata.2018.108 (2018).

**Publisher’s note**: Springer Nature remains neutral with regard to jurisdictional claims in published maps and institutional affiliations.

## Supplementary Material



Supplementary Information
