# Supplementary material for: Freshwater macroinvertebrate samples from a water quality monitoring network in the Iberian Peninsula
Source: Sci Data. 2018 Jun 5;5:180108. doi: 10.1038/sdata.2018.108 (PMC5987670; doi:10.1038/sdata.2018.108)
Supplement: Supplementary Information [file sdata2018108-s2.docx]

Table S1. List of the 64 standard terms used to map the information of the *Macroinvertebrate samples from the water quality monitoring network along the Ebro Basin* dataset. Definitions come from the Biodiversity Information Standards (<http://rs.tdwg.org/dwc/terms/index.htm>) [last accessed 23/01/2018]

| Extension | Terms | Definition |
| --- | --- | --- |
| Darwin Core Event | type | The nature or genre of the resource. For Darwin Core, recommended best practice is to use the name of the class that defines the root of the record. |
|  | modified | The most recent date-time on which the resource was changed. For Darwin Core, recommended best practice is to use an encoding scheme, such as ISO 8601:2004(E). |
|  | language | A language of the resource. Recommended best practice is to use a controlled vocabulary such as RFC 4646 [RFC4646]. |
|  | license | A legal document giving official permission to do something with the resource. |
|  | rightsHolder | A person or organization owning or managing rights over the resource. |
|  | institutionCode | The name (or acronym) in use by the institution having custody of the object(s) or information referred to in the record. |
|  | datasetName | The name identifying the data set from which the record was derived. |
|  | informationWithheld | Additional information that exists, but that has not been shared in the given record. |
|  | eventID | An identifier for the set of information associated with an Event (something that occurs at a place and time). May be a global unique identifier or an identifier specific to the data set. |
|  | samplingProtocol | The name of, reference to, or description of the method or protocol used during an Event. |
|  | sampleSizeValue | A numeric value for a measurement of the size (time duration, length, area, or volume) of a sample in a sampling event. |
|  | sampleSizeUnit | The unit of measurement of the size (time duration, length, area, or volume) of a sample in a sampling event. |
|  | samplingEffort | The amount of effort expended during an Event. |
|  | eventDate | The date-time or interval during which an Event occurred. For occurrences, this is the date-time when the event was recorded. Not suitable for a time in a geological context. Recommended best practice is to use an encoding scheme, such as ISO 8601:2004(E). |
|  | year | The four-digit year in which the Event occurred, according to the Common Era Calendar. |
|  | month | The ordinal month in which the Event occurred. |
|  | day | The integer day of the month on which the Event occurred. |
|  | verbatimEventDate | The verbatim original representation of the date and time information for an Event. |
|  | habitat | A category or description of the habitat in which the Event occurred. |
|  | eventRemarks | Comments or notes about the Event. |
|  | locationID | An identifier for the set of location information (data associated with dcterms:Location). May be a global unique identifier or an identifier specific to the data set. |
|  | continent | The name of the continent in which the Location occurs. Recommended best practice is to use a controlled vocabulary such as the Getty Thesaurus of Geographic Names. |
|  | waterBody | The name of the water body in which the Location occurs. Recommended best practice is to use a controlled vocabulary such as the Getty Thesaurus of Geographic Names. |
|  | country | The name of the country or major administrative unit in which the Location occurs. Recommended best practice is to use a controlled vocabulary such as the Getty Thesaurus of Geographic Names. |
|  | countryCode | The standard code for the country in which the Location occurs. Recommended best practice is to use ISO 3166-1-alpha-2 country codes. |
|  | stateProvince | The name of the next smaller administrative region than country (state, province, canton, department, region, etc.) in which the Location occurs. |
|  | municipality | The full, unabbreviated name of the next smaller administrative region than county (city, municipality, etc.) in which the Location occurs. Do not use this term for a nearby named place that does not contain the actual location. |
|  | locality | The specific description of the place. Less specific geographic information can be provided in other geographic terms (higherGeography, continent, country, stateProvince, county, municipality, waterBody, island, islandGroup). This term may contain information modified from the original to correct perceived errors or standardize the description. |
|  | verbatimLocality | The original textual description of the place. |
|  | verbatimElevation | The original description of the elevation (altitude, usually above sea level) of the Location. |
|  | verbatimCoordinates | The verbatim original spatial coordinates of the Location. The coordinate ellipsoid, geodeticDatum, or full Spatial Reference System (SRS) for these coordinates should be stored in verbatimSRS and the coordinate system should be stored in verbatimCoordinateSystem. |
|  | decimalLatitude | The geographic latitude (in decimal degrees, using the spatial reference system given in geodeticDatum) of the geographic center of a Location. Positive values are north of the Equator, negative values are south of it. Legal values lie between -90 and 90, inclusive. |
|  | decimalLongitude | The geographic longitude (in decimal degrees, using the spatial reference system given in geodeticDatum) of the geographic center of a Location. Positive values are east of the Greenwich Meridian, negative values are west of it. Legal values lie between -180 and 180, inclusive. |
|  | geodeticDatum | The ellipsoid, geodetic datum, or spatial reference system (SRS) upon which the geographic coordinates given in decimalLatitude and decimalLongitude as based. Recommended best practice is use the EPSG code as a controlled vocabulary to provide an SRS, if known. Otherwise use a controlled vocabulary for the name or code of the geodetic datum, if known. Otherwise use a controlled vocabulary for the name or code of the ellipsoid, if known. If none of these is known, use the value "unknown". |
|  | coordinateUncertaintyInMeters | The horizontal distance (in meters) from the given decimalLatitude and decimalLongitude describing the smallest circle containing the whole of the Location. Leave the value empty if the uncertainty is unknown, cannot be estimated, or is not applicable (because there are no coordinates). Zero is not a valid value for this term. |
| Darwin Core Occurrence | collectionCode | The name, acronym, coden, or initialism identifying the collection or data set from which the record was derived. |
|  | basisOfRecord | The specific nature of the data record. |
|  | occurrenceID | An identifier for the Occurrence (as opposed to a particular digital record of the occurrence). In the absence of a persistent global unique identifier, construct one from a combination of identifiers in the record that will most closely make the occurrenceID globally unique. |
|  | catalogNumber | An identifier (preferably unique) for the record within the data set or collection. |
|  | occurrenceRemarks | Comments or notes about the Occurrence. |
|  | recordedBy | A list (concatenated and separated) of names of people, groups, or organizations responsible for recording the original Occurrence. The primary collector or observer, especially one who applies a personal identifier (recordNumber), should be listed first. |
|  | organismQuantity | A number or enumeration value for the quantity of organisms. |
|  | organismQuantityType | The type of quantification system used for the quantity of organisms. |
|  | lifeStage | The age class or life stage of the biological individual(s) at the time the Occurrence was recorded. Recommended best practice is to use a controlled vocabulary. |
|  | establishmentMeans | The process by which the biological individual(s) represented in the Occurrence became established at the location. Recommended best practice is to use a controlled vocabulary. |
|  | preparations | A list (concatenated and separated) of preparations and preservation methods for a specimen. |
|  | disposition | The current state of a specimen with respect to the collection identified in collectionCode or collectionID. Recommended best practice is to use a controlled vocabulary. |
|  | identifiedBy | A list (concatenated and separated) of names of people, groups, or organizations who assigned the Taxon to the subject. |
|  | identificationReferences | A list (concatenated and separated) of references (publication, global unique identifier, URI) used in the Identification. |
|  | kingdom | The full scientific name of the kingdom in which the taxon is classified. |
|  | phylum | The full scientific name of the phylum or division in which the taxon is classified. |
|  | class | The full scientific name of the class in which the taxon is classified. |
|  | order | The full scientific name of the order in which the taxon is classified. |
|  | family | The full scientific name of the family in which the taxon is classified. |
|  | genus | The full scientific name of the genus in which the taxon is classified. |
|  | taxonRank | The taxonomic rank of the most specific name in the scientificName. Recommended best practice is to use a controlled vocabulary. |
|  | taxonRemarks | Comments or notes about the taxon or name. |
| Darwin Core Measurement Or Facts | measurementType | The nature of the measurement, fact, characteristic, or assertion. Recommended best practice is to use a controlled vocabulary. |
|  | measurementValue | The value of the measurement, fact, characteristic, or assertion. |
|  | measurementAccuracy | The description of the potential error associated with the measurementValue. |
|  | measurementUnit | The units associated with the measurementValue. Recommended best practice is to use the International System of Units (SI). |
|  | measurementDeterminedBy | The units associated with the measurementValue. Recommended best practice is to use the International System of Units (SI). |
|  | measurementMethod | A description of or reference to (publication, URI) the method or protocol used to determine the measurement, fact, characteristic, or assertion. |
|  | measurementRemarks | Comments or notes accompanying the MeasurementOrFact. |
